# Pre-Clinical Models of Diffuse Intrinsic Pontine Glioma

**DOI:** 10.3389/fonc.2015.00172

**Published:** 2015-07-24

**Authors:** Katherine L. Misuraca, Francisco J. Cordero, Oren J. Becher

**Affiliations:** ^1^Department of Pediatrics, Division of Hematology-Oncology, Duke University Medical Center, Durham, NC, USA; ^2^Department of Pathology, Duke University Medical Center, Durham, NC, USA

**Keywords:** brainstem glioma, DIPG, pre-clinical animal models

## Abstract

Diffuse intrinsic pontine glioma (DIPG) is a rare and incurable brain tumor that arises in the brainstem of children predominantly between the ages of 6 and 8. Its intricate morphology and involvement of normal pons tissue precludes surgical resection, and the standard of care today remains fractionated radiation alone. In the past 30 years, there have been no significant advances made in the treatment of DIPG. This is largely because we lack good models of DIPG and therefore have little biological basis for treatment. In recent years, however, due to increased biopsy and acquisition of autopsy specimens, research is beginning to unravel the genetic and epigenetic drivers of DIPG. Insight gleaned from these studies has led to improvements in approaches to both model these tumors in the lab and to potentially treat them in the clinic. This review will detail the initial strides toward modeling DIPG in animals, which included allograft and xenograft rodent models using non-DIPG glioma cells. Important advances in the field came with the development of *in vitro* cell and *in vivo* xenograft models derived directly from autopsy material of DIPG patients or from human embryonic stem cells. Finally, we will summarize the progress made in the development of genetically engineered mouse models of DIPG. Cooperation of studies incorporating all of these modeling systems to both investigate the unique mechanisms of gliomagenesis in the brainstem and to test potential novel therapeutic agents in a preclinical setting will result in improvement in treatments for DIPG patients.

## Introduction

Diffuse intrinsic pontine glioma (DIPG) arises in the pons of mostly children and is incurable. Of the 200–300 new cases of DIPG per year in the United States, the median age at diagnosis is 7 years of age, and the median survival is <1 year from diagnosis. Surgery is not an option, radiation provides only temporary relief, and no small molecule or chemotherapeutic agent has been demonstrated to prolong survival in this disease (1). Until very recently, the choice of drugs to evaluate in clinical trials for children with DIPG was based on pre-clinical studies in adult glioma models. This was primarily due to the lack of appreciation that DIPGs may respond differently to therapy than adult gliomas, as they are genetically distinct and arise in a different microenvironment, as well as the absence of genetically faithful DIPG pre-clinical models.

Pre-clinical models of glioma have been widely developed and used over the past several decades in order to study the initiation, progression, and treatment of adult and cerebral cortex gliomas. However, accumulating evidence of regional differences between DIPG and cerebral cortex glioma strongly suggests that they arise through distinct tumor driving mechanisms (2–8). Therefore, translating results of research from adult and cerebral cortex models to treatment strategies for DIPG has had little success. All DIPG treatment strategies, to date, including those based on the results of pre-clinical or clinical trials with cerebral cortex glioma, have failed to provide improved efficacy above the radiation standard of care (9). Consequently, a better understanding of DIPG tumor driving mechanisms is greatly needed in order to generate more accurate pre-clinical models of DIPG. Findings using these models will advance the translation of pre-clinical trials toward efficacious treatments for patients.

Historically, biopsy procedures were not performed on DIPG in humans due to the highly sensitive and critical nature of the brainstem, a tissue boasting very little functional redundancy with other regions of the brain. Given that surgical resection is not a part of the standard treatment regimen, acquisition of human DIPG specimens has been difficult. In recent years, however, several groups have shown the feasibility and safety associated with DIPG biopsy (10, 11), as well as the possibility of acquiring DIPG tissue at autopsy of sufficient quality for experimental purposes (12). This has not only begun to provide tumor specimens to directly generate more accurate cell and animal-based models but also provided much needed insight into the genetic alterations driving DIPG. This insight has allowed researchers the opportunity to genetically model DIPG in animals.

**Figure 1:**
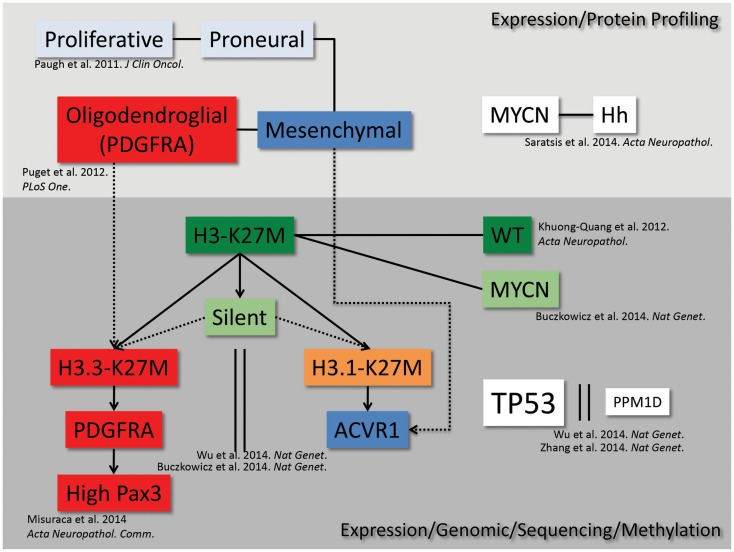
**DIPG subgrouping**. From high-throughput genetic, epigenetic, proteomic, and sequencing analyses, we map the current understanding of the interrelated subgroups within DIPG.

Recent gene expression, mutational, and epigenetic analyses of DIPG patient samples have revealed distinct subgroups present within the disease (Figure 1). The earliest studies analyzed gene expression of both autopsy and surgical patient samples and found that DIPG gene expression signatures clustered separately from non-brainstem pediatric high-grade gliomas, and gene set enrichment analysis revealed three distinct subgroups within DIPG (13). These gene expression subgroups were found to be significantly similar to the mesenchymal, proliferative, and proneural groups previously described in adult and non-brainstem pediatric high-grade glioma (14). Further studies have suggested additional subtypes of DIPG, characterized by upregulation of *MYCN*, Hedgehog, and *PDGFRA* that potentially overlap or fall outside of the original subgroups (15–18). Most recently, the identification of novel mutations in histone 3.3/3.1 (5, 8, 17) and *ACVR1* (18–21) further refined the characterization of DIPG subgroups. The identification of these subgroups and their respective genetic alterations calls for the development of new pre-clinical models to accurately represent the unique gene expression and epigenetic landscapes of DIPG that may impact therapeutic responses.

This review will detail the first attempts at modeling DIPG in animals, encompassing allograft and xenograft rodent models, as well as the development of *in vitro* systems and genetically engineered models. True improvements in the treatment of this disease will stem from the cooperation of studies incorporating all of these modeling systems.

## Transplantation-Based Rodent Models

Stereotactic implantation of glioma cells into the rodent brain has been a widely used tool for glioma research, although the development of models specifically in the brainstem has lagged behind those of the cerebral cortex. The first demonstration that heterotopic cells could grow in the rodent brainstem came from the injection of human medulloblastoma cells into the cisterna magna of nude rats, which led to tumor cell colonization in the medulla and pons (22). This suggested that modeling glioma in the brainstem of rodents might be a feasible experimental approach for studying the biology and treatment of DIPG, and led others to investigate this further using adult and neonatal rodents.

The first animal models developed for DIPG specifically involved the intracranial injection of rat glioma cell lines, F98, 9L, or C6 into the brainstem of neonatal (23, 24) or adult (25–27) rats leading to the generation of brainstem glioma (BSG). All of these allogenic orthotopic models utilized a stereotactic approach for implantation of the tumor cells into specific coordinates of the rat brain, targeting the pons. While these rats did develop tumors resembling gliomas in the appropriate location of the brainstem, the tumor cells had been derived from adult gliomas that arose in the cerebral cortex of rats and had been heavily passaged in culture. Therefore, although these models did take into account the specific microenvironment of the brainstem, any innate differences between cerebral cortex glioma and BSG cells were ignored.

Next, several groups generated human xenograft models in which human adult cerebral cortex glioblastoma cells, either from cell lines or serially transplanted xenografts, were transplanted into the brainstem of rats (28) or mice (29, 30), leading to tumors histologically and anatomically resembling human DIPG. As these tumors were composed of human glioma cells growing within the brainstem, these models were designed for the purpose of investigating therapeutic response rates, taking into account the unique microenvironment and blood–brain barrier of the brainstem. One murine xenograft model was used to test the effects of ionizing radiation (IR), the standard of care for DIPG, and found that escalating single doses of IR provided a temporary survival benefit, similar to what is seen in patients (29). Other studies incorporated bioluminescent imaging in order to show that treatment with chemotherapeutic agents, such as temozolomide (TMZ), or small molecule inhibitors like PD-0332991 (a CDK4/6 inhibitor) significantly delayed tumor growth in their xenograft models (28, 30). These results seem to suggest that TMZ might be suitable for patients with DIPG; however, clinical trials have not shown any efficacy (31, 32). This provides evidence that the use of glioma cells from the cerebral cortex, despite growing within the brainstem microenvironment, may not be suitable for predicting clinical response rates of DIPG.

## Human DIPG Cell and Xenograft Models

Given the limitations of the models discussed above, an important step in the field came when the first group developed human DIPG-specific cell and xenograft lines derived from autopsy material of a DIPG patient (33). DIPG autopsy tissue was cultured *in vitro* in neural stem cell conditions, which supports the growth of tumor neurospheres expressing (to varying degrees), Nestin, GFAP, Vimentin, Sox2, Olig2, and CD133, suggesting a primitive neural precursor cell type (33). Stereotactic transplantation of dissociated neurospheres into the fourth ventricle of immunodeficient neonatal mice led to the development of tumors in the hindbrain, diffusely infiltrating the brainstem, cerebellum, and cerebrum, with histopathology reminiscent of DIPG (33, 34). These were important studies, as they were the first to demonstrate a post-mortem cell culture strategy to generate *in vitro* neurosphere and *in vivo* xenograft DIPG models, similar to what has been well established for cerebral cortex glioma. These studies were followed by direct implantation of human DIPG cells from autopsy into mice without a prior culture step, also resulting in pontine DIPG-like tumors. However, these tumors, through an unknown mechanism, consisted of cells of murine as opposed to human origin (34).

These xenograft models can be used to evaluate novel treatment strategies for DIPG – for example, Sewing et al. investigated the treatment of autopsy-derived xenografts with the chemotherapeutic agent carmustine (BCNU) via convection-enhanced delivery (CED), showing that this delivery method may represent an effective drug delivery alternative for DIPG patients, bypassing the restrictive blood–brain barrier (35). One caveat of these models is that DIPG autopsy material has usually been subjected to radiation treatment as well as additional therapies, such as TMZ (a known mutagen), avastin, or other experimental agents prior to harvest, inducing genetic and epigenetic changes in the tumor cells that will impact therapeutic response.

In contrast to working with autopsy material, models of DIPG using tissue harvested from living biopsies are beginning to emerge as the practice of biopsy is becoming more common. Several groups have developed human DIPG cell lines from tumor samples harvested at diagnosis during biopsy procedures (36, 37). These cell lines were used to test the effects of the targeted agents dasatinib and cabozantinib *in vitro* (36), as well as the combination of radiation and the Wee1 inhibitor MK-1775 (38). In addition, these human cell lines can be used to study the biology of DIPG, as was done by Chan et al. who studied the methylation and gene expression pattern changes induced by the H3K27M mutation in tumor cells (39).

Others have implanted DIPG cells collected from biopsy into mice after *in vitro* neurosphere culture (37, 40). Specifically, Thirant et al. injected the cells into the striatum rather than the brainstem, and Hashizume et al. modified the cells prior to injection by infecting with hTERT and a luciferase reporter. The model generated by Hashizume et al. was characterized as expressing GFAP, Nestin, Olig2, and PDGFRα, similar to human DIPG. This model was subjected to gene expression and copy number analysis to compare it to previous analyses of human DIPG samples (37), and was used as a preclinical tool to test the *in vivo* effects of radiation therapy combined with MK-1775 (38). More recently, Grasso et al. (41) used both autopsy and biopsy patient-derived DIPG cells to generate *in vitro* and *in vivo* model systems. This study screened 83 drugs and focused on the histone deacetylase inhibitor panobinostat, one of the most potent drugs *in vitro*. Importantly, Grasso et al. found treatment with panobinostat alone significantly reduced orthotopic xenograft growth and synergized *in vitro* with the histone de-methylase inhibitor GSK-J4, a drug, which had been previously shown to be efficacious in both cell and xenograft DIPG models (42).

These studies provide important precedent that naïve DIPG cells from biopsy specimens are able to grow *in vitro* and be propagated in immunocompromised mice *in vivo*. Future work in this system should be expanded upon to test potential therapeutic agents or delivery mechanisms. Although biopsy tissue represents a small sample of the tumor and may not be representative of the entire tumor, these types of models will perhaps be more predictive of therapeutic response rates in patients than models based on autopsy material, as the cells have not been previously subjected to treatment. Regardless, these human DIPG-derived cell and xenograft models are much improved over the initial allograft rodent models, as they are composed of human DIPG cells and are growing within the unique microenvironment of the brainstem. The studies described herein using these models exemplify how xenograft systems can further our understanding of the mechanisms underlying DIPG tumorigenesis and can be used as a pre-clinical tool to test potential therapeutics.

## Genetically Engineered Models of DIPG

An important complement to human xenografts in mice is the use of genetically engineered models, as these systems are driven by a specific set of genetic alterations introduced in a particular cell-of-origin. The establishment of such models was delayed, however, by a lack of knowledge regarding the genetic drivers of DIPG. The increased understanding in recent years of the underlying genetics of DIPG provides a starting point for genetically modeling these tumors in the lab.

**Figure 2:**
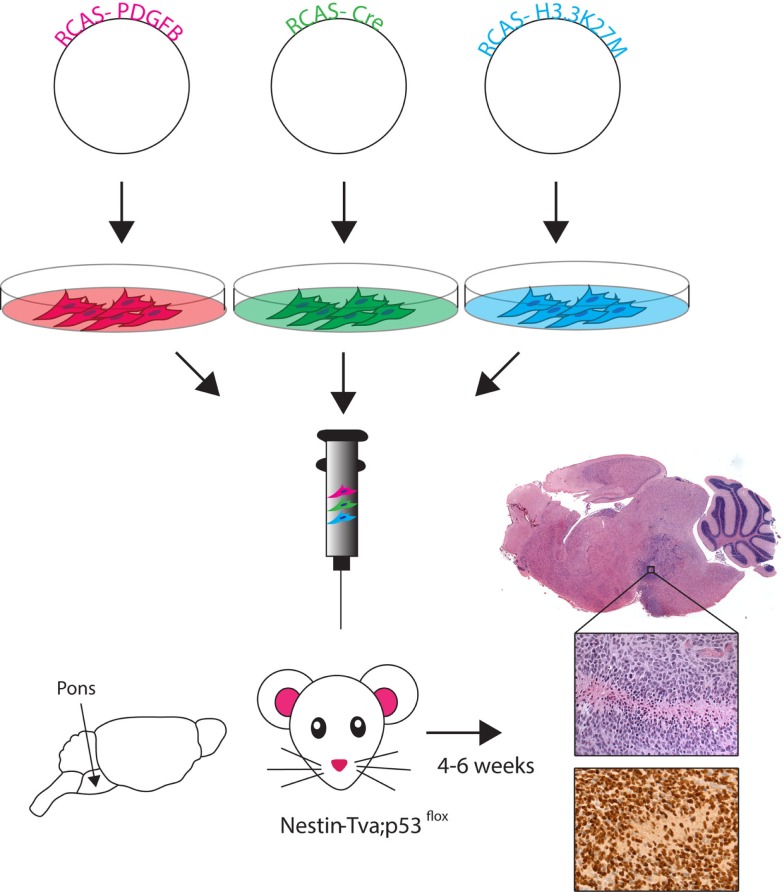
**RCAS-based modeling of DIPG**. Schematic of the RCAS-based GEMM. Individual RCAS plasmids are transfected into virus producing cells. These cells are then directly injected into the brainstem of neonatal (Postnatal day 3–4) mice in an even ratio after stable virus production begins. Viruses selectively infect Nestin-expressing cells lining fourth ventrical of the brainstem. Tumor symptoms (e.g., loss of balance, enlarged head) develop within 4–6 weeks. Histology using hemotoxylin and eosin (H&E) staining shows ventral brainstem location of tumor. High magnification (40×) H&E image reveals high-grade tumor characteristics (pseudopalisading necrosis, mitotic figures, neovascularization), and immunohistochemistry image shows the HA-tagged H3.3K27M (nuclear) and PDGFB (cytoplasmic) expressing cells.

Genetically engineered mouse models (GEMMs) provide the advantage of studying tumors that arise in their natural microenvironment in immune-proficient animals. The first GEMMs of DIPG were generated using the RCAS (replication-competent avian sarcoma-leucosis virus long-terminal repeat with splice acceptor)/tumor virus A (TVA) modeling system and genetic alterations commonly found in the human disease (Figure 2) (43–45). The RCAS–Tva system uses the retroviral avian leucosis and sarcoma virus family as a gene delivery vector. This virus exclusively infects cells expressing the corresponding surface receptor TVA, normally expressed in avian cells. Genetically engineered mice have been generated to express the TVA receptor under the control of several cell-type specific promoters (46). Using these mice, the RCAS system revealed gliomas could be generated targeting cells outside of the subventricular zone (47). This finding showed the potential of the system to be used for modeling DIPG.

Early work with the RCAS system used germline Ink4a-ARF loss and PDGFB overexpression targeted to Nestin-expressing cells of the neonatal mouse pons (44), the likely cells-of origin for DIPG (33). Tumors arising in this model were located within the murine brainstem and were histologically similar to human DIPG; however, they were not exclusive to the pons region of the brainstem. Therefore, this system is described as a BSG GEMM and additional, more spatiotemporally accurate, models are needed for DIPG. Identification of pons-specific promoters or enhancers would allow for the generation of mouse strains that drive expression of Tv-a (for use with the RCA/Tv-a system), Cre, or DIPG-specific oncogenes. Alternatively, pons-derived stem cells can be isolated, cultured, and infected by RCAS viruses *in vitro*, and re-introduced into the murine pons as an *ex vivo* approach although the *in vitro* culture conditions will alter the cells.

Becher et al. (44) was the first to use GEMMs to generate high-grade BSGs and test for therapeutic response using radiation and perifosine, an inhibitor of AKT signaling. They found a survival benefit using a 10 Gy radiation dose; however, no additional benefit was seen using the combined radiation with perifosine treatment. However, another study using this early GEMM did demonstrate a survival benefit with the CDK4/6 inhibitor PD-0332991 alone and in combination with radiation (43). Importantly, this was the first demonstration of any targeted agent prolonging survival over radiation therapy alone in a pre-clinical setting. This early BSG GEMM showed that modeling glioma in the mouse brainstem was possible and was pivotal in the identification of a promising new therapy for DIPG.

RCAS-based GEMMs have evolved by utilizing the increasing understanding of DIPG genetic alterations, incorporating the three most highly recurring genetic alterations: PDGF signaling overexpression, p53 loss, and the H3.3K27M mutation (45). Cells producing the RCAS-PDGFB, RCAS-Cre, and RCAS-H3.3K27M viruses were delivered into the brainstem of Nestin-Tva; p53-floxed neonatal mice and resulted in high-grade BSG with gene expression similar to human data (Figure 2) (45). This model was the first to show global loss of H3K27me3 levels with expression of the K27M mutation, similar to what is seen in patient samples. The DIPG pre-clinical consortium recently used this GEMM to test the promising new drug BMS-754807, a potent multi-kinase inhibitor (48). So far, no survival benefit has been found using the novel BSG GEMM, but Halvorson et al. showed promising *in vitro* data and found the drug concentration levels in treated mice were well below the known IC-50 for BMS-754807. The aggressiveness of this most recent BSG GEMM parallels the clinical disease and represents a good tool for pre-clinical therapeutic trials.

In addition to being used as a pre-clinical tool to test potential therapeutics for DIPG, GEMMs can be used to study the biology of the disease in an *in vivo* setting, which may help to uncover novel characteristics of the human disease. In this regard, using the RCAS model described above, comparison of gene expression profiles of BSGs versus those initiated in the cerebral cortex revealed that those in the brainstem harbored high levels of the transcription factor Pax3 (49). *In vitro* and *in vivo* studies suggest that overexpression of this gene contributes to the gliomagenesis process by inhibiting apoptosis and promoting proliferation. Analysis of human tumor cohorts showed that 40% of DIPG patients’ tumors are characterized by particularly high levels of Pax3, which associates with activation of PDGF signaling, amplification of cell cycle regulatory genes, and is exclusive of ACVR1 mutations. This work further defines a subset of human DIPG (Figure 1) and lends insight into novel mechanisms driving tumorigenesis.

An additional genetic model recently used human embryonic stem cells to generate neural precursor-like cells, which were then transformed with activated PDGFRα, H3.3K27M, and p53 knockdown (50). With these transformed cells, Funato et al. were able to study the effects of these oncogenes both *in vitro* and after transplantation into the mouse pons, and found them to cooperate with one another to induce tumorigenesis. Although the cell-of-origin used in these studies may not represent a true *in vivo* cell of origin for DIPG, this study importantly illustrated the potential for using data from genomic and expression analysis to model the genetic drivers of DIPG *in vivo* using human-derived embryonic stem cells.

Although recently much progress has been made in the genetic modeling of DIPG in mice, additional models are needed in order to more accurately reflect the human disease. As discussed above, identification of promoters or enhancers specific to the neonatal pons will allow for the generation of more spatially accurate tumors. In addition, the RCAS/Tv-a GEMMs discussed herein effectively model the PDGFRA/oligodendroglial/H3.3K27M subgroup of DIPG (Figure 1). Utilization of alternative RCAS drivers, such as H3.1K27M, ACVR1 mutants, or MYCN, is needed in order to generate accurate models of other subtypes of the disease, identify underlying biological differences between different subtypes, and identify subtype-specific therapeutic strategies. Finally, the current GEMMs for DIPG are based on a neonatal cell-of-origin; however, we cannot rule out the possibility that DIPG originates *in utero*. Thus, introduction of DIPG genetic alterations to mice *in utero* via viral infection (including RCAS viruses into Tv-a transgenic mice) or electroporation [potentially combined with rising new technologies, such as the Piggybac transposon system (51)] could lend valuable insight into the effects of DIPG driver expression before birth.

## Conclusion

Pre-clinical models of DIPG are continuing to improve as our knowledge of DIPG etiology expands. The current models each have advantages to their use and have all contributed invaluable insight into DIPG biology and treatment (Table 1). Human cell and xenograft models have the benefit of being derived directly from DIPG patients. Cell lines derived from autopsy are likely altered by treatment, and thus, biopsy-derived cell lines, although rare, are biologically superior for identifying novel agents to treat newly diagnosed tumors. Treatment-related alterations found in autopsy samples, however, are valuable tools, which, when compared to pre-treatment tissue may provide insight into the changes occurring in DIPG cells in response to therapy, including metabolic changes, additional mutations, and the expansion of tumor subpopulations. These observations could provide both explanations for the failure of attempted therapies and strategies for combating therapeutic resistance.

**Table 1:** **Animal models of DIPG**.

Model	Rationale	Method	Description	Reference
F98, 9L, and C6 rat brainstem glioma	Use of rodent cerebral glioma cell lines to create tumors in the brainstem	Allosteric, orthotopic, stereotactic injection	Rodent cortical glioma cell lines transplanted into the rat or mouse pons	(23–27)
Cerebral xenograft	Human cerebral glioma cells used to generate tumors in rodent brainstem	Stereotactic injection of adult cerebral cortex glioma cells	Patient-derived cortical glioblastoma cells are introduced into the rodent brainstem	(28–30)
DIPG Xenograft	Autopsy or biopsy-derived patient DIPG cells used to propagate murine tumors for drug testing	Stereotactic injection of human DIPG cells	Autopsy and biopsy-derived patient DIPG cells are used for injection into rodent brainstem	(33–39, 41)
DIPG cell lines	DIPG patient cells cultured *in vitro* for use in drug screening and general research	Isolated human DIPG cells cultured in stem cell conditions	Autopsy and biopsy-derived patient DIPG cells are cultured *in vitro* under stem/progenitor conditions	(33, 34, 36–39, 41)
RCAS GEMMs	Use of transgenic mice with introduced genetic alterations found in DIPG as pre-clinical models	Genetically engineered mouse models using retroviral gene delivery targeting the mouse brainstem	Transgenic mice expressing RCAS viral receptor under cell-type-specific promoters are infected with oncogenes important in DIPG	(41, 43, 44, 46, 47)
Human embryonic stem cells	Development of human derived puripotent stem cells for modeling DIPG	Human embryonic stem cells are differentiated to a neuronal progenitor lineage and co-transduced with common DIPG alterations	*In vitro* derived neuronal progenitors are transduced with constitutively active PDGFRA, p53 shRNA, and H3.3K27M for *in vitro* analysis or murine implantation	(50)

Xenografts of human cells into immunocompromised mice also retain the advantage of using patient-derived cells. However, full recapitulation of human tumor development is compromised by the alteration of tumor microenvironment and absence of an immune system. Therefore, GEMMs are an important complement to human xenograft models, and while they have shown much promise, they remain underdeveloped. The next generation of GEMMs should incorporate the knock-in of common DIPG mutations, such as H3.3K27M, into the endogenous loci in order to better simulate the expression level and localization of the mutated gene products. Additionally, while current GEMMs use overexpression of PDGFB ligand as an oncogenic driver, amplification of the wild-type PDGFRα receptor is most common in patients (13). Therefore, a GEMM incorporating amplification of PDGFRα will be another improvement for future development, as will models incorporating oncogenic drivers found in the other subtypes of DIPG.

The increased development and availability of cell models, xenografts, and GEMMs of DIPG represent great promise to improve the treatment of this deadly disease. While the path from development of genetically faithful animal models to generation of efficacious therapies can be long and arduous, continuing to improve the models as more information about the human disease is uncovered is necessary. Using the available tools to identify novel therapeutic agents and causes of therapy resistance will be paramount in developing effective treatment strategies. Cooperation between researches utilizing all of the available pre-clinical models discussed here will be the key to advancement in this field. These models represent valuable tools, which will aid in testing future therapeutic agents and lend further insight into the biological mechanisms of DIPG.

## Conflict of Interest Statement

The authors declare that the research was conducted in the absence of any commercial or financial relationships that could be construed as a potential conflict of interest.
